# Seroepidemiological Study of Canine and Human Dirofilariasis in the Endemic Region of Northern Serbia

**DOI:** 10.3389/fvets.2020.00571

**Published:** 2020-09-29

**Authors:** Sara Savić, Marina Zekic Stosic, Doroteja Marcic, Isabel Hernández, Aleksandar Potkonjak, Suzana Otasevic, Maja Ruzic, Rodrigo Morchón

**Affiliations:** ^1^Scientific Veterinary Institute “Novi Sad”, Novi Sad, Serbia; ^2^Group of Animal and Human Dirofilariasis, Faculty of Pharmacy, Campus Miguel Unamuno, University of Salamanca, Salamanca, Spain; ^3^Department of Veterinary Medicine, Faculty of Agriculture, University of Novi Sad, Novi Sad, Serbia; ^4^Faculty of Medicine, University of Nis, Nis, Serbia; ^5^Clinic for Infectious Diseases, Faculty of Medicine, Clinical Center of Vojvodina, University of Novi Sad, Novi Sad, Serbia

**Keywords:** *Dirofilaria immitis*, *Dirofilaria repens*, Serbia, dogs, humans, prevalence, seroprevalence, Europe

## Abstract

Dirofilariasis is a vector-borne zoonotic disease caused mainly by *Dirofilaria immitis* and *Dirofilaria repens* that affect dogs and humans all over the world. Serbia is considered an endemic country to both forms of dirofilariasis, although most of the population is concentrated in the north of the country. The aims of this study were to show the prevalence of *D. immitis* and *D. repens* in dogs and the seroprevalence in humans compared to previous studies in Northern Serbia. In total, 346 dog sera samples and 265 human samples were analyzed. Dog blood samples were analyzed using the modified Knott's method to check whether there were *Dirofilaria* spp. microfilariae and serum samples were checked by a commercial *D. immitis* antigen test. Human serum samples were analyzed with a non-commercial ELISA for detection of specific anti-*D. immitis*, anti-*D. repens*, and anti-*Wolbachia* IgG antibodies, and confirmed by western blotting. The overall prevalence for *Dirofilaria* spp. in dogs was 29.19%. The overall prevalence for *D. immitis* was 26.30%. The percentages of *D. immitis* and *D. repens* microfilaremia in dogs were 25.72 and 1.45%, respectively, while *D. immitis*./*D. repens* microfilaremia co-infections were also 1.45%. The overall seroprevalence for *Dirofilaria* spp. in humans was 3.77%. The overall seroprevalence for *D. immitis* was 1.51, 1.13% for *D. repens*, and for *D. immitis*/*D. repens* co-infections was 1.13%. The results indicate that *D. immitis* and *D. repens* are present in dogs and humans in the province of Vojvodina, in the northern part of Serbia. It is most likely associated with the presence of many rivers, the climate, and presence of mosquitoes in the area, so there could be a real public health risk.

## Introduction

Dirofilariasis is a vector-borne zoonotic disease caused mainly by *Dirofilaria immitis* and *Dirofilaria repens*. *Dirofilaria immitis* causes heartworm disease in canines and pulmonary dirofilariasis in humans, whereas *D. repens* causes canine subcutaneous dirofilariasis and ocular/subcutaneous dirofilariasis in humans. Both parasites are transmitted by culicid mosquitoes, which inoculate larva 3 into definitive hosts in both animals and humans. For that reason, dirofilariasis is considered a veterinary and public health problem (1, 2).

Canine heartworm disease is a chronic, progressive, and life-threatening disease in which adult worms stay in the pulmonary artery and the heart, in the right ventricle of definitive hosts. In canine subcutaneous dirofilariasis, the adult worms are usually beneath the skin forming a subcutaneous nodule. In both cases, microfilariae circulate in the blood stream and are ingested by several species of mosquito vectors during their blood-feeding (3) and after two successive molts, during the next blood meal (4), stage-3 larvae are inoculated into the definitive host. In humans, *D. immitis* immature worms cause embolization in the pulmonary microarteries, leading to the formation of benign lung nodules (pulmonary dirofilariasis), although most cases are asymptomatic (1). On the other hand, *D. repens* worms do not usually reach the adult stage and immature worms may cause larva migrans syndrome and form subcutaneous nodules, in the ocular region and other organs (1, 3, 5–7). Pulmonary dirofilariasis usually has no clinical symptoms, so most diagnostic tools cannot be used, making it much more difficult to identify. However, subcutaneous/ocular dirofilariasis presents clinical signs that are easier to detect (1, 3). Moreover, *D. immitis* and *D. repens* harbor endosymbiotic bacteria of the genus *Wolbachia*. This bacteria participates in the parasite's life cycle and embryogenesis and plays a key role in the immune and inflammatory response of the organism to the disease (4, 8–10).

Dirofilariasis is on the rise in the European population of dogs and humans (1, 3, 11, 12). It is considered to be an endemic disease in southern European countries and in central and northern countries such as Switzerland, Germany, Netherlands, Lithuania, Slovenia, Czech Republic, Slovakia, and Russia (2, 4, 13–18). In addition, in the last decade, different epidemiological and seroepidemiological studies, alongside clinical reporting, have shown that dirofilariasis has been introduced into the countries of the Balkans peninsula (1, 3, 4, 11, 19). Serbia is considered as an endemic country to both forms of dirofilariasis in dogs (20–24). Few human cases have been reported to be caused by *D. repens* or have had specific antibodies found (25, 26). To explain the rise of dirofilariasis, studies suggest that red foxes and golden jackals may serve as reservoir hosts (27) and *Culex pipiens* and *Aedes vexans* act as vectors of both diseases in Northern Serbia (28).

The aim of this study was to show the prevalence of *D. immitis* and *D. repens* in dogs and the seroprevalence in humans compared to previous studies in Northern Serbia.

## Materials and Methods

### Study Area

The northern part of Serbia (Province of Vojvodina) lies between Hungary, Croatia, and Romania. This northern part of the country is largely plains with a continental climate and a lot of rivers. Summers are hot and have lengthened over time due to climate change, so temperatures over 14°C usually last even through to the end of October. Winters are less and less cold and there has not been much snow during the last several winters. All this means that the mosquito season is prolonged from March to October. The air humidity during the warm period of the year is mostly high, meaning that the conditions for the development of mosquitoes are appropriate. During springtime there is a lot of rain and often in some parts of the country there are floods.

In the province of Vojvodina there is the Danube river, which crosses the country from east to west, while the Tisa river flows from north to south, and there are smaller rivers all around the region (**Figure 2**). There is also an artificial canal system called Danube–Tisa–Danube Canal. It covers the total area in Vojvodina of about 12,700 km^2^ and it consists of a number of canals. This Canal is a unique hydro-engineering system for flood control and hydrotechnical management, forestry, water supply, wastewater evacuation, navigation, tourism, fishing, and hunting. Besides these purposes, it also represents a substantial amount of water, convenient for development of mosquitos.

### Serum Samples

We analyzed a total of 611 sera samples: 346 dog sera samples (173 male and 173 female) were obtained from dogs analyzed by several veterinary clinics (Table 1) and 265 human samples (208 male and 57 female) were provided to the research laboratory from different departments of clinical centers in Northern Serbia (Table 2). Human samples were taken from patients with different symptoms, not only those with specific symptoms that could point to dirofilariasis. Variables considered for the analyses were gender, age, and municipality of residence. Samples were collected preserving the privacy of the patients and informing them of how the samples would be used. All samples were collected during the period of 2018 to 2019.

**Table 1:** Distribution of prevalence of *D. immitis, D. repens*, and co-infections in dogs in Northern Serbia by gender and age.

		***D. immitis***		***D. repens***		***D. immitis/D. repens***		***Dirofilaria* spp**.
	**Dog samples *n*^**°**^ (%)**	**Test Positive (%)**	**Knott Positive (%)**	**Test Positive (%)**	**Knott Positive (%)**	**Test Positive (%)**	**Knott Positive (%)**	**TOTAL Positive (%)**
**Gender**								
Male	173 (50.00%)	54 (31.21%)	52 (30.06%)	No test	1 (0.58%)	2 (1.16%)/No test	2 (1.16%)	57 (32.95%)
Female	173 (50.00%)	37 (21.39%)	37 (21.39%)	No test	4 (2.31%)	3 (1.73%)/No test	3 (1.73%)	44 (25.43%)
**Age**								
<3	162 (46.82%)	44 (27.16%)	44 (27.16%)	No test	1 (0.62%)	0 (0.00%)/No test	0 (0.00%)	45 (27.78%)
3–5	133 (38.44%)	34 (25.56%)	34 (25.56%)	No test	2 (1.50%)	2 (1.50%)/No test	2 (1.50%)	38 (28.57%)
6–8	30 (8.67%)	11(36.66%)	9 (30.00%)	No test	1 (3.33%)	2 (6.67%)/No test	2 (6.67%)	14 (46.66%)
>9	21 (6.07%)	2 (9.52%)	2 (9.52%)	No test	1 (4.76%)	1 (4.76%)/No test	1 (4.76%)	4 (19.05%)
Total	346	91 (26.30%)	89 (25.72%)	No test	5 (1.45%)	5 (1.45%)/No test	5 (1.45%)	101 (29.19%)

**Table 2:** Distribution of seroprevalence of *D. immitis, D. repens*, and co-infections in humans in Northern Serbia by gender and age.

	**Human samples n^**°**^ (%)**	***D. immitis***	***D. repens***	***D. immitis/D. repens***	***Dirofilaria* spp**.
		**ELISA Positive (%)**	**ELISA Positive (%)**	**ELISA Positive (%)**	**TOTAL Positive (%)**
**Gender**					
Male	208 (78.49%)	4 (1.92%)	3 (1.44%)	2 (1.44%)	9 (4.33%)
Female	57 (21.51%)	0	0	1 (1.75%)	1 (1.75%)
**Age**					
<20	2 (0.75%)	0	0	0	0 (0.00%)
20–40	48 (18.11%)	2 (4.17%)	0	1 (2.08%)	3 (6.25%)
>40	215 (81.13%)	2 (0.93%)	3 (1,40%)	2 (0.93%)	7 (3.25%)
Total	265	4 (1.51%)	3 (1.13 %)	3 (1.13 %)	10 (3.77%)

### Methods

Dog blood samples were analyzed by applying the modified Knott's technique (29) to check whether there were *Dirofilaria* spp. microfilariae in the blood of the animals included in the study. Morphological characteristics of microfilariae (cephalic and caudal ends) were used in order to differentiate *D. immitis* and *D. repens* microfilariae (30). Dog serum samples were tested for the presence of *D. immitis* adult antigens using a commercial immunochromatographic test kit (VetLine *Dirofilaria* Antigen, NovaTec, Germany) according to the manufacturer's instructions. There is no commercial laboratory test of any kind for *D. repens* in dogs.

Human serum samples were analyzed using a non-commercial ELISA for detection of specific anti-*D. immitis*, anti-*D. repens*, and anti-*Wolbachia* IgG antibodies with some modifications (16, 17, 31, 32). *D. immitis* and *D. repens* adult worm extracts (DiSA and DrSA, respectively) and 1:100 serum dilutions were used to detect the presence of anti-DiSA and DrSA IgG antibodies. Sera samples diluted at 1:40 with a recombinant form of the *Wolbachia* Surface Protein (rWSP) were used to detect the presence of anti-rWSP IgG antibodies. In both cases, goat anti-human IgG (H+L) conjugated to horseradish peroxidase (Sigma-Aldrich, Spain) was used at a 1:4000 dilution. Easy Reader (Bio-Rad laboratories, USA) was used for measuring optical densities (OD) at 492 nm. The cut-off point (OD = 0.8 for DiSA and DrSA and 0.5 for rWSP) was determined by calculating the mean value + 3 standard deviations (3SD) of 50 serum samples obtained from dogs and clinically healthy humans (negative controls) who belonged to an area free of *D. immitis* and *D. repens*. When both non-commercial ELISAs gave positive results for the same serum sample, that human sera were considered positive. Additionally, by using western blot analysis performed according to a previously described methodology (16, 33–36), these results were confirmed. Both antigenic extracts were subjected to SDS–PAGE in 12% gels under reduced conditions, and proteins were transferred onto nitrocellulose. Human sera were analyzed at a 1:40 dilution and anti-conjugates at a 1:500 dilution. All samples that were positive with these kits were also analyzed by western blot to determine if they recognized the specific bands for *D. immitis* (17–22 kDa) and for *D. repens* (43–70 kDa).

### Geographical Information System

The ArcGIS Pro online software was used for the construction of a map of the sampling area. All layers of relevant environmental information (rivers, irrigated croplands, natural parks, among others) were included and symbolized for a better understanding of the map. All dog and human samples infected with *D. immitis, D. repens*, and co-infections were manually georeferenced by GPS at the point of capture. Georeferenced positive data for both hosts are shown in the map.

### Statistical Analysis

The SPSS Base 18.0 software for Windows was used for the data analysis. The descriptive analysis of the considered variables was carried out studying the proportions in the qualitative variables. To compare proportions, Chi-square tests were performed. In all the cases, the significance level was established at *p* < 0.05.

## Results

The overall prevalence for *Dirofilaria* spp. in dogs was 29.19% (Table 1). The overall prevalence for *D. immitis* was 26.30%. The percentage of *D. immitis* microfilaremia in dogs was 25.72% (in all cases with a positive *D. immitis* antigen test), 1.45% for *D. repens* microfilaremia, and 1.45% for *D. immitis*/*D. repens* microfilaremia co-infections (in all cases with a positive *D. immitis* antigen test). There are significant differences between the prevalence for *D. immitis* infected male and female dogs with a higher prevalence in male dogs, whereas the prevalence of *D. repens* was higher in females (*p* < 0.05).

**Figure 1:**
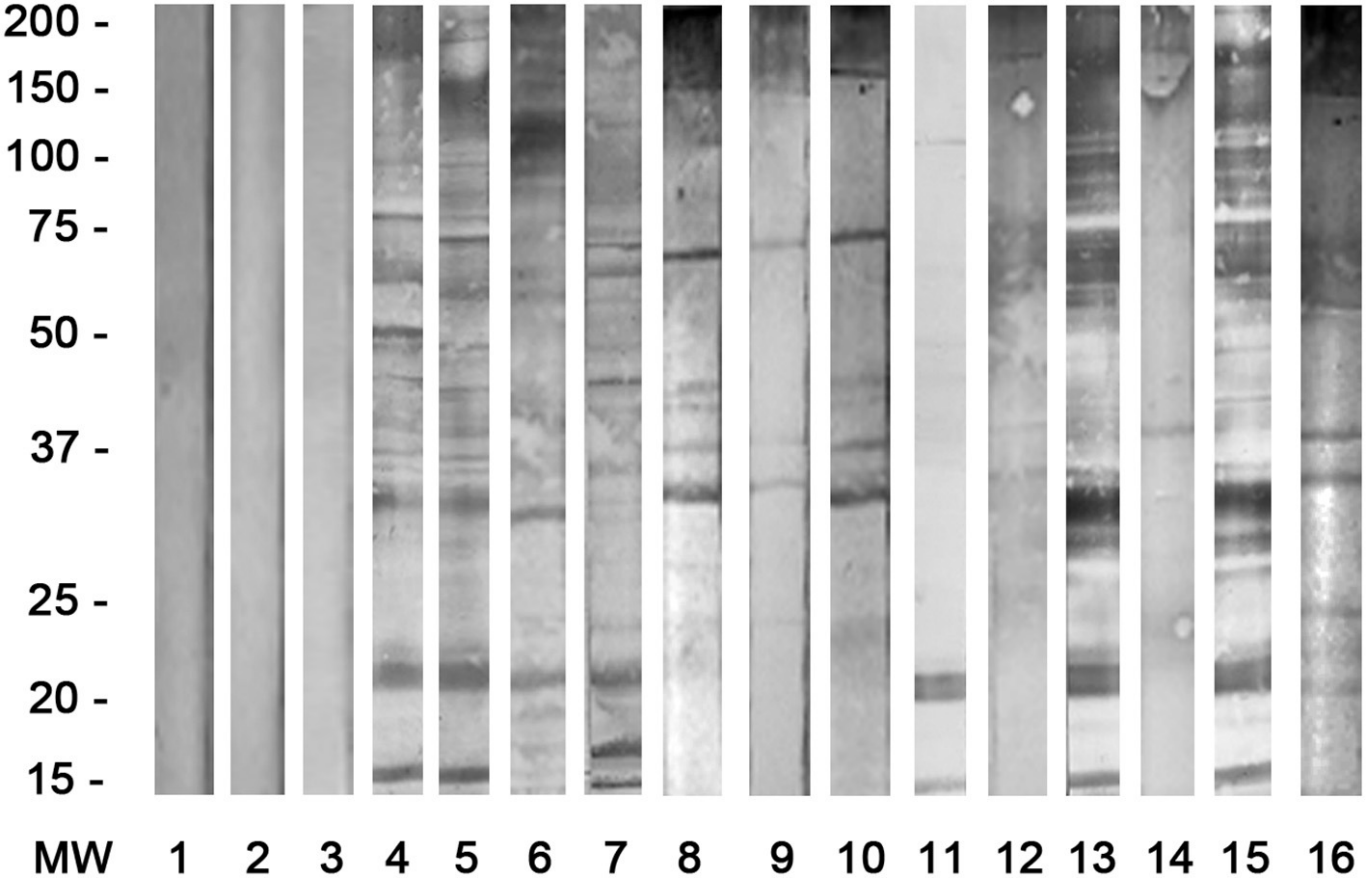
Western blot in all human seropositive cases for *D. immitis* (4–7) with specific bands at 17–22 kDa; for *D. repens* (8–10) with bands at 43–70 kDa; for co-infections *D. immitis/D. repens* (11/12, 13/14, 15/16), and negative sera (1–3).

The overall seroprevalence for *Dirofilaria* spp. in humans was 3.77%. These results are shown in Table 2. The overall seroprevalence for *D. immitis* was 1.51, 1.13% for *D. repens*, and 1.13% for *D. immitis*/*D. repens* co-infections. All positive cases were detected in males with significant differences (*p* < 0.05) for *D. immitis* (1.92%) and *D. repens* (1.44%), but not in co-infection (1.44%). All positive samples via western blot analysis are shown in Figure 1.

By age, there are significant differences between the seroprevalences of *D. immitis* between the 20–40 range and the other ranges, the seroprevalences of *D. repens* between the over-40 age group and the other ranges and the seroprevalences of co-infections between the 20–40 age group and the other ranges (*p* < 0.05).

**Figure 2:**
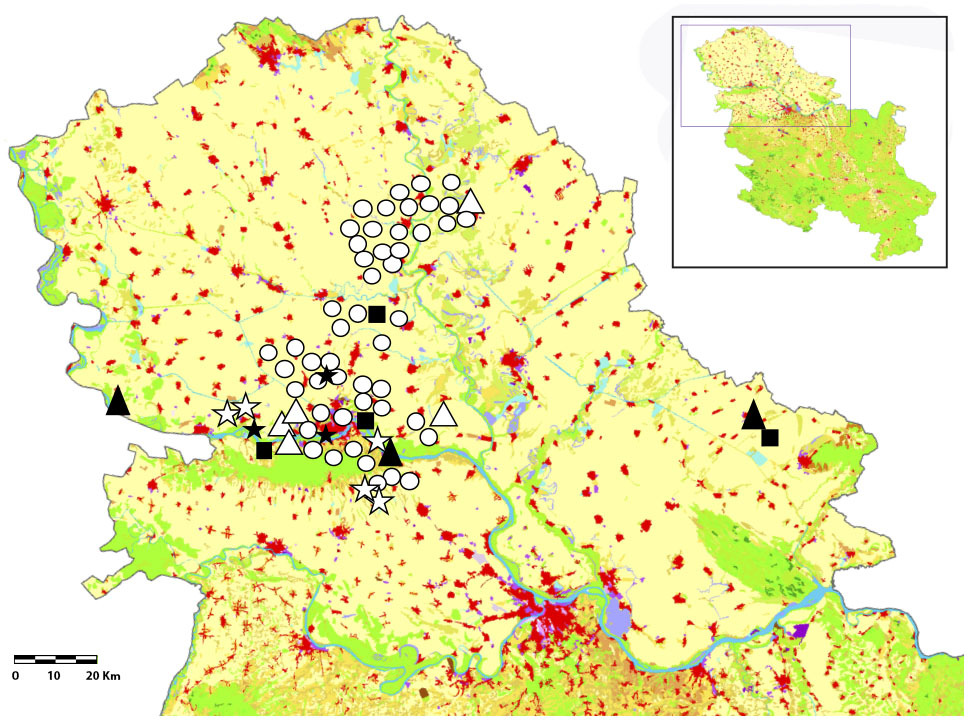
Localization of the *D. immitis* and *D. repens* positive dogs and humans and co-infections in Northern Serbia: *D. immitis* in dogs (O), *D. repens* in dogs (Δ), *D. immitis/D. repens* in dogs (⋆), and *D. immitis* in humans (■), *D. repens* in humans (▴), and *D. immitis/D. repens* in humans (⋆). In green (natural grasslands, mixed forest, broad-leaved forest, moors and heathland, parks, forest parks), in red (continuous and discontinuous urban areas) and in blue (rivers, areas of stagnant water, and water bodies).

Regarding the geolocation of the positive samples of both dogs and people on a map (Figure 2), all of them were located in the vicinity of rivers, forest parks, green areas, and even within some cities in northern Serbia. There are only two cases of humans infected with *D. immitis* and *D. repens* in the northeast of the country.

## Discussion

Wherever canine dirofilariasis exists, there is a risk of human infection. The general climatic conditions, local environmental factors, human interventions on the environment, and pet management are also factors that determine the distribution and incidence of illness (36). Humans and *Dirofilaria* spp. species have developed limited mutual adaptation (1). L3s inoculated by mosquitoes are usually eliminated from the host by the immune system. However, an undetermined percentage survives and continues to develop until pre-adult, and in some cases of *D. repens*, until adult, causing pulmonary and subcutaneous nodules or sometimes in the eye area, encapsulated or non-encapsulated. In any case, contact with the infecting larvae and subsequent stages of development stimulates an immune response that can be measured with appropriate techniques (37).

Epidemiological studies of human dirofilariasis, unlike in the dog population, have followed two different approaches. One is reported retrospective reviews and the other is seroepidemiological analyses. Each of these approaches provides information on different yet complementary aspects of human infections. The information obtained through retrospective reviews of reported cases offer only a partial view, since it only includes the part of the affected population that develops some type of clinical manifestation, and regions of endemicity showing vectors with zoo-anthropophilic habits probably have higher frequencies of human infections than reported in the literature. The problem of underreporting may exist due to the fact that symptoms in dirofilariasis patients, especially in pulmonary infections, may be misdiagnosed or unnoticed (1). Seroepidemiological studies compliment this information by detecting contact through the measurement of anti-*Dirofilaria* antibodies, allowing the evaluation of the risk of dirofilariasis infection in a defined geographical region, and constituting an excellent measure of the risk of infection for the human population which resides in an endemic area. Seroepidemiological studies of residents of areas of endemicity reported higher rates of infection in a similar manner to those of canines of the same areas (1, 5, 13, 16, 17, 34).

The aim of the present study was to analyze the prevalence in dogs and the response to anti-*D. immitis* and/or anti-*D. repens* antibodies in the northern region of Serbia (Vojvodina), taking into account that this region has been considered endemic for some time. Furthermore, the aim was to identify the potential risk of infection of the human population in an endemic area.

In Serbia, humidity and temperature conditions during a large proportion of the year allow for the transmission of dirofilariasis, with seroepidemiological data revealing noteworthy prevalence rates in the country's dog populations and human clinical cases caused by both *D. immitis* and *D. repens* (1, 3, 24).

In the current study, a prevalence of 26.30% was observed in dogs infected by *D. immitis*, with 25.72% microfilaremia in the total population, and the presence of *D. repens* larvae in 1.45% of the analyzed dogs. In two regions of Vojvodina (Pancevo and Veliko Gradiste), the previously reported seroprevalence was 22.9% for *D. immitis* and the presence of *D. repens* microfilariae was 39.34% (38). In Bulgaria and Croatia, two neighboring countries, there are studies about the prevalence of *D. immitis* and *D. repens* in dogs (8.1 and 11.1%, respectively) and co-infections (3.2%) (11). Several studies point to an increase in *D. immitis* infections and a decrease of an infection with *D. repens* in recent years (21–23) which is corroborated by results in this study. In addition, infections have also been found to be prevalent in wild canids (39), which could mean there is a risk of *D. immitis* infection between the dog population and the wild canid population. Meanwhile, *D. repens* was found to circulate mostly in golden jackal and red fox populations (27).

With regards to the human population, the seroprevalence for *Dirofilaria* spp. in humans was 3.77, 1.51% for *D. immitis*, 1.13% for *D. repens*, and 1.13% for *D. immitis*/*D. repens* co-infections. This is the first time these tests have been conducted in this region of Serbia. Human cases originating from *D. repens* have only previously been reported in the region of southeastern Serbia (24, 40, 41). In addition, other studies have reported seroprevalences of 9.7% and 8.1% against *D. repens* and D. immitis polyproteins specific antibodies, respectively, and 2.3% in individuals with specific antibodies to both species (26). Similar studies in neighboring countries such as Romania and Moldova have reported seroprevalences of 10.7% for *D. immitis*, 0.2% for *D. repens*, and 0.9% for both parasites (16). In addition, in Croatia there have been human *D. repens* cases reported (3). These seroepidemiological studies are a good tool to measure the risk of infection in a population where there is a high population of infected animals, as well as the presence of vectors, which serve as a vehicle for transmission of the disease (1, 42).

Both animals and infected people were geolocated in the immediate vicinity or in a relatively close environment of potential mosquito breeding areas, which poses a risk to those areas. The two cases of humans infected by *D. immitis* and *D. repens* in the northeast of the country are close to cities where cases of dogs and humans had already been reported (38) and were located in areas near rivers and green areas where mosquitoes breed. The spatial distribution of positive cases has a clear association with different geo-environmental factors, humidity and temperature, the existence of irrigated areas, areas with abundant water, rivers in valleys protected from winds, and proximity to the coast, which are considered risk factors for the transmission of dirofilariasis (43).

In conclusion, the results indicate that *D. immitis* and *D. repens* are present in dogs and humans in the province of Vojvodina, in the northern part of Serbia. It is most probably associated with the presence of many rivers, the climate, and the presence of mosquitoes, so there is a real public health risk. Serology studies in humans can be very useful for indicating the exposure to *Dirofilaria* spp. in a healthy population in order to obtain useful data on the epidemiological scenario of human dirofilariasis in Serbia and in Europe. That exposure was confirmed in the current study. Further studies addressing the control of dirofilariasis in the dog population are needed to reduce the risk of infection in the human population.

## Data Availability Statement

All datasets generated for this study are included in the article/supplementary material.

## Ethics Statement

Ethical approval was not provided for this study on human participants because People data don't require permission, everyone has verbal consent. Written informed consent to participate in this study was provided by the participants' legal guardian/next of kin. Ethical review and approval was not required for the animal study because the blood samples were taken during a regular blood checkup of the dogs and the consent was gained from the owners so there was no need for the ethical approval. Written informed consent for participation was not obtained from the owners because Verbal consent of client owned dogs.

## Author Contributions

SS and RM designed the study and wrote the manuscript. MS, DM, IH, AP, SO, and MR performed the fieldwork, collected the data, and performed the experiments. All authors participated in the discussion of the results, corrected, read, and approved the final manuscript.

## Conflict of Interest

The authors declare that the research was conducted in the absence of any commercial or financial relationships that could be construed as a potential conflict of interest. The reviewer AG declared a past co-authorship with one of the authors SS to the handling editor.

## References

[1] Simón F Siles-Lucas M Morchón R González-Miguel J Mellado I Carretón E. Human and animal dirofilariasis: the emergence of a zoonotic mosaic. Clin Microbiol Rev. (2012) 25:507–44. 10.1128/CMR.00012-12 22763636 PMC3416488

[2] Genchi C Kramer L. Subcutaneous dirofilariasis (*Dirofilaria repens*): an infection spreading throughout the old world. Parasit Vectors. (2017) 10:517. 10.1186/s13071-017-2434-8 29143643 PMC5688444

[3] Capelli G Genchi C Baneth G Bourdeau P Brianti E Cardoso L. Recent advances on *Dirofilaria repens* in dogs and humans in Europe. Parasit Vectors. (2018) 11:663. 10.1186/s13071-018-3205-x 30567586 PMC6299983

[4] Morchón R Carretón E González-Miguel J Mellado-Hernández I. Heartworm disease (Dirofilaria immitis) and their vectors in Europe - new distribution trends. Front Physiol. (2012) 3:196. 10.3389/fphys.2012.00196 22701433 PMC3372948

[5] Kartashev V Batashova I Kartashov S Ermakov A Mironova A Kuleshova Y. Canine and human dirofilariasis in the rostov region (southern Russia). Vet Med Int. (2011) 2011:685713. 10.4061/2011/685713 21253482 PMC3022198

[6] Ilyasov B Kartashev V Bastrikov N Morchón R González-Miguel J Simón F. Delayed diagnosis of dirofilariasis and complex ocular surgery, Russia. Emerg Infect Dis. (2013) 19:326–8. 10.3201/eid1902.121388 23460991 PMC3559065

[7] Ilyasov B Kartashev V Bastrikov N Madjugina L González-Miguel J Morchón R. Thirty cases of human subcutaneous dirofilariasis reported in Rostov-on-Don (Southwestern Russian Federation). Enferm Infecc Microbiol Clin. (2015) 33:233–7. 10.1016/j.eimc.2014.04.002 24948573

[8] Kramer LH Tamarozzi F Morchón R López-Belmonte J Marcos-Atxutegi C Martín-Pacho R. Immune response to and tissue localization of the Wolbachia surface protein (WSP) in dogs with natural heartworm (*Dirofilaria immitis*) infection. Vet Immunol Immunopathol. (2005) 6:303–8. 10.1016/j.vetimm.2005.03.011 15876457

[9] Grandi G Morchón R Kramer L Kartashev V Simón F. Wolbachia in *Dirofilaria repens*, an agent causing human subcutaneous dirofilariasis. J Parasitol. (2008) 94:1421–3. 10.1645/GE-1575.1 19127968

[10] Simón F Kramer LH Román A Blasini W Morchón R Marcos-Atxutegi C. Immunopathology of *Dirofilaria immitis* infection. Vet Res Commun. (2007) 31:161–71. 10.1007/s11259-006-3387-0 17216316

[11] Farkas R Mag V Gyurkovszky M Takács N Vörös K Solymosi N. The current situation of canine dirofilariasis in Hungary. Parasitol Res. (2020) 119:129–35. 10.1007/s00436-019-06478-5 31754854 PMC6942023

[12] Velev V Vutova K Pelov T Tsachev I. Human Dirofilariasis in Bulgaria between 2009 and 2018. Helminthologia. (2019) 56:247–51. 10.2478/helm-2019-0016 31662696 PMC6799580

[13] Kartashev V Tverdokhlebova T Korzan A Vedenkov A Simón L González-Miguel J. Human subcutaneous/ocular dirofilariasis in the Russian Federation and Belarus, 1997-2013. Int J Infect Dis. (2015) 33:209–11. 10.1016/j.ijid.2015.02.017 25722281

[14] Diosdado A Gómez PJ González-Miguel J Simón F Morchón R. Current status of canine dirofilariasis in an endemic area of western Spain. J Helminthol. (2018) 92:520–3. 10.1017/S0022149X17000591 28669358

[15] Miterpáková M Valentová D Cabanová V Berešíková L. Heartworm on the rise-new insights into *Dirofilaria immitis* epidemiology. Parasitol Res. (2018) 117:2347–50. 10.1007/s00436-018-5912-9 29774422

[16] Ciuca L Simón F Rinaldi L Kramer L Genchi M Cringoli G. Seroepidemiological survey of human exposure to *Dirofilaria* spp. in Romania and Moldova. Acta Trop. (2018) 187:169–74. 10.1016/j.actatropica.2018.07.012 30056075

[17] Fontes-Sousa AP Silvestre-Ferreira AC Carretón E Esteves-Guimarães J Maia-Rocha C Oliveira P. Exposure of humans to the zoonotic nematode *Dirofilaria immitis* in northern Portugal. Epidemiol Infect. (2019) 147:e282. 10.1017/S0950268819001687 31793429 PMC6805740

[18] Sabunas V Radzijevskaja J Sakalauskas P Petkevičius S Karveliene B Žiliukiene J. *Dirofilaria repens* in dogs and humans in Lithuania. Parasit Vectors. (2019) 12:177. 10.1186/s13071-019-3406-y 30999960 PMC6472076

[19] Ionică AM Matei IA D'Amico G Ababii J Daskalaki AA Sándor AD. Filarioid infections in wild carnivores: a multispecies survey in Romania. Parasit Vectors. (2017) 10:332. 10.1186/s13071-017-2269-3 28705255 PMC5508779

[20] Savić-Jevdenić S Vidić B Grgić Ž Milovanović A Brza dijagnostika dirofilarioze pasa u regionu Novog Sada. Vet Glasnik. (2004) 58:693–8.

[21] Tasić A Rossi L Tasic-Otasevic S Miladinovic-Tasic N Ilic T Dimitrijevic S. Survey of canine dirofilariasis in Vojvodina, Serbia. Parasitol Res. (2008) 103:1297–302. 10.1007/s00436-008-1132-z 18712415

[22] Spasojević-Kosić LJ Lalošević V Lalošević D Simin S Vasić I Kuruca LJ Prevalence of dirofilariasis in pet dogs in Novi Sad. Contemp Agric. (2012) 61:247–54.

[23] Spasojević-Kosić LJ Lalošević V Simin Kuruca LJ Dirofilariasis and Angiostrongilosis in pet and hunting dogs in Novi Sad, Vojvodina, Serbia. Arhiv Vet Med. (2016) 9:53–62. 10.46784/e-avm.v9i2.89

[24] Krstić M Gabrielli S Ignjatović M Savić S Cancrini G Randelović G. An appraisal of canine and human cases reveals an endemic status of dirofilariasis in parts of Serbia. Mol. Cell Probes. (2017) 31:37–41. 10.1016/j.mcp.2016.08.005 27539018

[25] DŽamić AM Colović IV Arsić-Arsenijević VS Stepanović S Boričić I DŽamić Z. Human *Dirofilaria repens* infection in Serbia. J Helminthol. (2009) 83:129–37. 10.1017/S0022149X09341346 19379543

[26] Tasić-Otašević SA Gabrielli SV Tasić AV Miladinovićtasić NL Kostić JT Ignjatović AM. Seroreactivity to *Dirofilaria antigens* in people from different areas of Serbia. BMC Infect Dis. (2014) 14:68. 10.1186/1471-2334-14-68 24507413 PMC3922270

[27] Potkonjak A Rojas A Gutiérrez R Nachum-Biala Y Kleinerman G Savić S. Molecular survey of *Dirofilaria* species in stray dogs, red foxes and golden jackals from Vojvodina, Serbia. Comp Immunol Microbiol Infect Dis. (2020) 68:101409. 10.1016/j.cimid.2019.101409 31881413

[28] Kurucz K Kepner A Krtinic B Zana B Földes F Bányai K. First molecular identification of *Dirofilaria* spp. (Onchocercidae) in mosquitoes from Serbia. Parasitol Res. (2016) 115:3257–60. 10.1007/s00436-016-5126-y 27193348

[29] Acevedo RA Theis JH Kraus JF Longhurst WM. Combination of filtration and histochemical stain for detection and differentiation of *Dirofilaria immitis* and *Dipetalonema reconditum* in the dog. Am J Vet Res. (1991) 42:537–40. 7196718

[30] Genchi G Venco L Genchi M Guideline for the laboratory diagnosis of canine and feline *Dirofilaria* infections. In: Genchi C, Rinaldi L, Cringoli G, editors. Mappe Parassitologighe 8, Dirofilaria immitis and Dirofilaria repens in dog and cat and human infection, eds. Rolando Editore, Salamanca (2007). p. 137–45.

[31] Simón F Muro A Cordero M Martin J. A seroepidemiologic survey of human dirofilariasis in Western Spain. Trop Med. arasitol. (1991) 42:106–8. 1896765

[32] Simón F Prieto G Morchón R Bazzocchi C Bandi C Genchi C. Immunoglobulin G antibodies against the endosymbionts of filarial nematodes (Wolbachia) in patients with pulmonary dirofilariasis. Clin Diagn Lab Immunol. (2003) 10:180–1. 10.1128/CDLI.10.1.180-181.2003 12522059 PMC145277

[33] Perera L Muro A Cordero M Villar E Simón F. Evaluation of a 22kDa *Dirofilaria immitis* antigen for the immunodiagnosis of human pulmonary dirofilariasis. Trop Med Parasitol. (1994) 45:249–52. 7899798

[34] Perera L Pérez-Arellano JL Cordero M Simón F Muro A. Utility of antibodies against a 22 kD molecule of *Dirofilaria immitis* in the diagnosis of human pulmonary dirofilariasis. Trop Med Int Health. (1998) 3:151–5. 10.1046/j.1365-3156.1998.00209.x 9537278

[35] Santamaría B Cordero M Muro A Simón F. Evaluation of *Dirofilaria immitis* excretory/secretory products for seroepidemiological studies on human dirofilariasis. Parasite. (1995) 2:269–73. 8520802 10.1051/parasite/1995023269

[36] Simon F Prieto G Muro A Cancrini G Cordero M Genchi C. Human humoral immune response to *Dirofilaria* species. Parassitologia. (1997) 39:397–400. 9802100

[37] Carretón E Morchón R Montoya-Alonso JA Chapter 1. Dirofilariasis cardiopulmonar canina. In: Montoya-Alonso JA, Carretón E, editors. Dirofilariasis. Pautas de Manejo Clínico. Barcelona: Multimédica Ediciones Veterinarias (2012). p. 1–130.

[38] Tasić A Tasić-Otašević S Gabrielli S Miladinović-Tasić N Ignjatović A Dordević J. Canine Dirofilaria infections in two uninvestigated areas of Serbia: epidemiological and genetic aspects. Vector Borne Zoonot Dis. (2012) 12:1031–5. 10.1089/vbz.2011.0949 23127188 PMC3525891

[39] Cirović D Penezić A Pavlović I Kulišić Z Cosić N Burazerović J. First records of Dirofilaria repens in wild canids from the region of central Balkan. Acta Vet Hung. (2014) 62:481–8. 10.1556/avet.2014.021 25410390

[40] Dzamić AM Arsić-Arsenijević V Radonjić I Mitrović S Marty P Kranjcić-Zec IF. Subcutaneous *Dirofilaria repens* infection of the eyelid in Serbia and Montenegro. Parasite. (2004) 11:239–40. 15224588

[41] Tasić S Stoiljković N Miladinović-Tasić N Tasić A Mihailović D Rossi L. Subcutaneous dirofilariasis in south-east Serbia - case report. Zoonoses Public Health. (2011) 58:318–22. 10.1111/j.1863-2378.2010.01379.x 21740534

[42] Cabrera E Carretón E Morchón R Falcón-Cordón Y Falcón-Cordón S Simón F. The Canary Islands as a model of risk of pulmonary dirofilariasis in a hyperendemic area. Parasitol Res. (2018) 117:933–6. 10.1007/s00436-018-5774-1 29396676

[43] Simón L Afonin A López-Díez LI González-Miguel J Morchón R Carretón E. Geo-environmental model for the prediction of potential transmission risk of Dirofilaria in an area with dry climate and extensive irrigated crops. The case of Spain. Vet Parasitol. (2014) 200:257–4. 10.1016/j.vetpar.2013.12.027 24456900

